# Atlanta Society of Medicine

**Published:** 1896-12

**Authors:** L. Amster, M. B. Hutchins, W. L. Champion, R. R. Kime, F. W. McRae

**Affiliations:** Atlanta, Ga.; Atlanta, Ga.; Atlanta, Ga.; Atlanta, Ga.; Atlanta, Ga.


### SOCIETY REPORTS.

### ATLANTA SOCIETY OF MEDICINE.

ATLANTA, GA., November 17, 1896.

The meeting was called to order by the President, Dr. R. R. KIME.

The following members were present: Drs. Pinckney, Elkin, Kime, Sommerfield, Hancock, Hutchins, Hardon, Collins, Stephens, Divine, Stockard, Champion, Amster, Grandy, Dunbar Roy, Richardson, Gilbert, and Arch Avary.

The minutes of the previous meeting were read and approved.

DR. W. S. ELKIN made some remarks upon the treatment of sprained ankle by adhesive strips, and exhibited the method of applying them. See page 662.

DR. DUNBAR ROY read an interesting and elaborate paper upon Nasal Syphilis.

Report of the committee appointed at last meeting to consider and suggest ways and means of increasing interest in and attendance upon meetings of the Society was then called for. By way of preface to this report, Dr. AMSTER, Chairman of the ommittee, made the following remarks:

"Some weeks ago I received a notice from the Secretary of our Society, that there would be a meeting to consider certain important changes of our constitution and by-laws, to determine the advisability of dividing the Society into sections, etc., and there would also be read a very interesting paper. The Secretary, in order to bring about a large attendance at this important meeting, inserted notices in our daily papers, and I naturally anticipated seeing a good assembly of our professional brethren. There were not more than a handful of doctors present. Why, you couldn't coax the members to come to one of your deliberations if you were to tempt them to an intellectual feast of nectar and ambrosia.

"Fortunately we counted a quorum at that meeting. In the course of our deliberations this little band of humble doctors, still true to their standard, aroused by the supreme indifference of the numerous members of this Society, commenced the sowing of a seed, which in time shall sprout and bloom and bear fruit, and make this Association an important factor in medical circles of this city, this State, and the South to which it is justly entitled.

"There must be something radically rotten in Denmark, that hundreds of physicians in Atlanta cannot and will not spare the time to attend two evenings a month, in which to meet their professional brethren, to foster good will and harmony, and to contribute to science their little share.

"The diligent perusal of current medical literature and the faithful attendance at Society meetings are acknowledged to be the two most powerful factors and educators of the professional man after leaving the medical schools. Have we, in Atlanta, reached the *ne plus ultra* of knowledge and the pinnacle of wisdom? Have we, in Atlanta, nothing more to learn, nothing to teach, that we can afford to dispense with the great institution of Medical Associations?

"If the good and well-meaning portion of our fraternity, the high-minded gentlemen who habitually walk the ethical path of professional righteousness, are willing to dispense with the many benefits and pleasures that are to be derived from scientific discussions and social affiliations, it is at least their duty to unite and protect themselves from the many charlatans and quacks who have infested the city, to the detriment of the legitimate practitioner and the woe of the unsophisticated, humbug-loving public. It is the duty of the legitimate physician to cry 'Halt!' to a lot of unscrupulous men who, disguised under the cloak of respectability, commit outrageous offenses against our time-honored code, commit daily breaches, not only against ethics, but against honest principle. They demoralize public trust and confidence in our art and science, and offer their incompetent services for sale for considerations which the ordinary day laborer would scorn with contempt.

"Gentlemen, I have proof positive that there are doctors in this city who hire themselves to families, consisting of six and eight members, for the monthly consideration of one dollar, making sometimes ten to twenty visits for that paltry sum. Can a young, struggling medicus, who is willing to pursue an honorable course, work against such tremendous odds? These philanthropic doctors do not discriminate between rich and poor. They contract themselves for one dollar a month to people who are able to pay double that amount for a single visit.

"Five dollars for an obstetrical case they consider a princely remuneration. Almost daily I am offered obstetrical engagements at that sum from people who are able to pay the customary fee. They claim they can get Dr. So-and-So for five dollars a case. Only recently I saw one of these cut-rate labor cases, which Dr. Cheapjohn left bleeding for six weeks after confinement. Shall we not protect ourselves and the public from such men, who tend to lower the dignity of our profession? Shall such men participate in equal privileges with the regular practitioner? They must be made to come to terms, or be shown up in their true light. Their privileges must be restricted to such an extent that no regular physician shall contaminate himself by giving aid and assistance to them. Debar them from the rights of consultation, and force them to do right from necessity if they fail to do it from instinct.

"Another point to be considered is the regulating of fees for life insurance examinations. Why do great life insurance companies, doing business of many millions of dollars every year, cut the fees of the medical examiner, upon whose integrity and efficiency their very life, prosperity and existence depends? Because of the many Cheapjohn doctors.

"To enlarge upon the gross misconduct of some members of the profession against each other, in their daily intercourse, would be entirely superfluous. We all know they are of frequent occurrence. We all know what methods some resort to for personal gain.

"I do not hesitate to say that the older and wiser members of the profession are largely responsible that such little interest is taken in the affairs of this Association and its scientific deliberations. Largely to them is due the lack of legislation to regulate the practice of medicine in this city and to enforce strict adherence to the universally adopted code of ethics. The young doctor naturally emulates the example of his older brother, the junior is willing to be instructed by his senior.

"Have you ever thought of it, you older members, why so many patients leave this city for Northern hospitals and institutions and to consult Northern doctors? If you older practitioners and teachers and proprietors of sanitariums would come to well-attended meetings of the Association, air your knowledge, and listen to the remarks of the younger members, impress them with the idea of your capability, the young doctors would not allow their sick to rush North, and let a class of patients—which we should mostly strive to retain—fill the coffers of Northern celebrities.

"Gentlemen, I can cite an instance, and three well-known surgeons will bear me out in my statement, that a gentleman of some means would not trust his little girl to have a glandular abscess opened. He wanted to take her to Baltimore, and it took four doctors to convince him that it could be done in Atlanta. The very same gentleman took his wife North, spending a large sum for an operation which could have been done here as well.

"I have as yet to see a case that left the city that could not have been as well attended here.

"Where is there a better opportunity for mutual understanding, and to know one another more intimately, socially and professionally, than the meeting of our Medical Society? Atlanta, the queen city of the South, where every branch of industry and commerce and science is forging to the front, cannot rise above the level of mediocrity in medicine and surgery. Why is it that men of the South—and she has furnished some of the most brilliant and shining lights—have to go North to gain recognition and fame? Is Southern soil not fertile enough to foster medical scientists? Are we simply 'visitors' of the sick? Cannot we also gain laurels in our art?

"It is indeed sad to contemplate when we compare our negligence with the activity of the North and foreign lands. How their medical societies flourish; how they vie with each other to discover new truths, and make new conquests in their chosen field, and we in Atlanta, cannot even support one medical society, and stimulate thought and knowledge to the best of our ability.

"I really do wonder why our local society of medicine is such a poor, forsaken institution?

"Some say there are too many cliques, that the members of the faculties of the different medical colleges are too antagonistic. Some say they are too busy lecturing and practicing medicine. Some love their home comforts too much after the day's labor is done. Some, I am told, are so liberally blest in this world's goods, that they do not care to inconvenience themselves any more. Some, they say, consider themselves so wise and perfect that there is nothing to be profited by attending the medical society meetings. Some believe there is not enough attraction. No library, no journals, etc. Some younger members do not attend meetings because the older members do not participate, and do not care to meet always the same monotonous few. Some are not satisfied with the present meeting place, etc.

"My opinion is that all these accusations are mere slander. That the medical fraternity of Atlanta, from some inexplicable cause, has fallen into a state of prolonged apathy—to use a celebrated phrase, it has fallen into a state of 'innocuous desuetude.' I say that the inactivity of the members is simply slumbering, and it is only necessary to sound the bugle, and all will awaken to their sense of duty, and will fall into line.

"It only remains for me to urge the members of the society and the profession at large to give aid and assistance and to co-operate with the committee appointed to provide ways and means whereby it will be possible to stimulate the usefulness and to reform and rejuvenate the Atlanta Society of Medicine.

"If necessary, compulsory membership and compulsory attendance at meetings ought to be adopted. Conscientious censors ought to be elected who will rigidly enforce strict adherence to ethics, etc. What at first will seem compulsion, will eventually become a pleasure and recreation to each and all of us.

"If my remarks appear unkind and exaggerated to you, I beg indulgence. My only aim is to see this society in a flourishing condition, and I hope to see the day, when it will be considered an honor to be a member of the Atlanta Society of Medicine, and that we will soon be able to point with just pride to the work we are capable of accomplishing."

The following report of the committee was then submitted:

Mr. President: The committee appointed to recommend ways and means to revive interest in affairs of the Atlanta Society of Medicine, beg leave to make the following report:

After mature and earnest consideration, we think it feasible and practicable to amend our constitution and by-laws in the following manner:

First. No member of this Society shall consult or recognize as a regular practitioner of medicine any physician who shall have become a resident practitioner for six months, and shall have failed to become a member of this Society, or whose membership shall have ceased from any cause.

Second. Any active member who, for three consecutive months, shall fail to attend the regular meetings without a good and sufficient reason (to be determined by the Society), shall be dropped from the roll.

Third. The Society shall elect a committee of three to act as a Board of Censors, whose duties shall be to take cognizance of overt breaches of the code of ethics; shall receive notices from the members of the Society of such breaches under seal of confidence; shall call attention of members charged with the offense to the fact of such charges, and if satisfactory settlement is not agreed upon by the Board of Censors and the accused, then the whole matter is to be referred to the Society for final adjustment. It shall also be the duty of the Board of Censors to locate and prosecute offenders against existing State laws. The Society shall have the power to remove any member of the Board of Censors who shall be found negligent in the performance of his duties.

Fourth. The Society shall have the power to discipline its members for immoral or unprofessional conduct, by reprimand and suspension, or expulsion from membership; to regulate the fee bills, and to decide all questions of ethics that may arise among its members.

We further recommend the early adoption of a fee bill, the distribution of printed copies of the fee bill among the members, who shall post the same in a conspicuous place in their offices.

We also urge upon the Society to provide headquarters and a meeting place, to be paid for from the funds of the Association; also to devise means for the establishment of a library.

Respectfully submitted.

L. AMSTER,

M. B. HUTCHINS,

W. L. CHAMPION,

R. R. KIME,

F. W. MCRAE.

